# Complexity in disguise: a systematic review of fractal analysis in psychiatric neuroimaging

**DOI:** 10.1007/s00330-026-12630-4

**Published:** 2026-05-26

**Authors:** Marta Reales-Moreno, Alexandra Korda, Stefan Borgwardt

**Affiliations:** 1 Department of Medical Sciences (School of Medicine), University of Girona, Girona, Spain; 2 Department of Psychiatry and Psychotherapy, University of Lübeck, Lübeck, Germany; 3 Department of Psychology, Cornell University, Ithaca, NY USA

**Keywords:** Brain patterns, Fractals, MRI, Neuroimaging, Psychiatry

## Abstract

**Objectives:**

Psychiatric diagnosis and fractal studies are complex processes that extend beyond clinical evaluation and require careful methodological considerations in neuroimaging. Over the years, fractals have helped reduce these complexities in research, but they still cannot grant clinical diagnoses. Thus, the main objective was a systematic review exploring the potential applications of fractal analysis in characterizing psychiatric conditions through neuroimaging techniques—including both functional and structural MRI.

**Materials and methods:**

A systematic literature review was conducted on PubMed, identifying thirty-nine original studies that met the inclusion criteria. Areas showing statistical significance (*p* < 0.05) were reported. These studies were categorized according to DSM-V classification and examined for the description of psychiatric conditions through the fractal analysis.

**Results:**

The review primarily focuses on young adults with psychiatric conditions compared to control groups. Schizophrenia and Autism Spectrum Disorder are major areas of investigation, and fractal dimension (FD) is the primary analysis method used to reflect brain patterns. Studies that calculated whole-brain FD may have underestimated local abnormalities due to the inclusion of a high percentage of tissue, potentially resulting in overlooked findings. Notably, abnormalities in the frontal cortex represent a common neurobiological feature across several psychiatric conditions.

**Conclusions:**

The findings from this systematic review shed light on the use of fractal analysis to quantify complex brain patterns in both psychiatric patients and healthy individuals. However, it is essential to recognize the need for further research to elucidate a fractal analysis protocol that allows for optimal extraction of psychiatric insights.

**Key Points:**

***Question*** *Fractal analysis applied to structural and functional MRI help characterize brain alterations across psychiatric conditions*.

***Findings*** *This review shows consistent fractal patterns across multiple psychiatric disorders, especially in frontal regions. Despite heterogeneous methodologies, results highlight shared structural and functional abnormalities*.

***Clinical relevance*** *Fractal analysis may offer complementary characterization of subtle brain organization across psychiatric disorders. Its potential clinical utility—such as improving diagnostic characterization, earlier detection, among others—remains limited by the current absence of a standardized protocol*.

**Graphical Abstract:**

**Figure:**
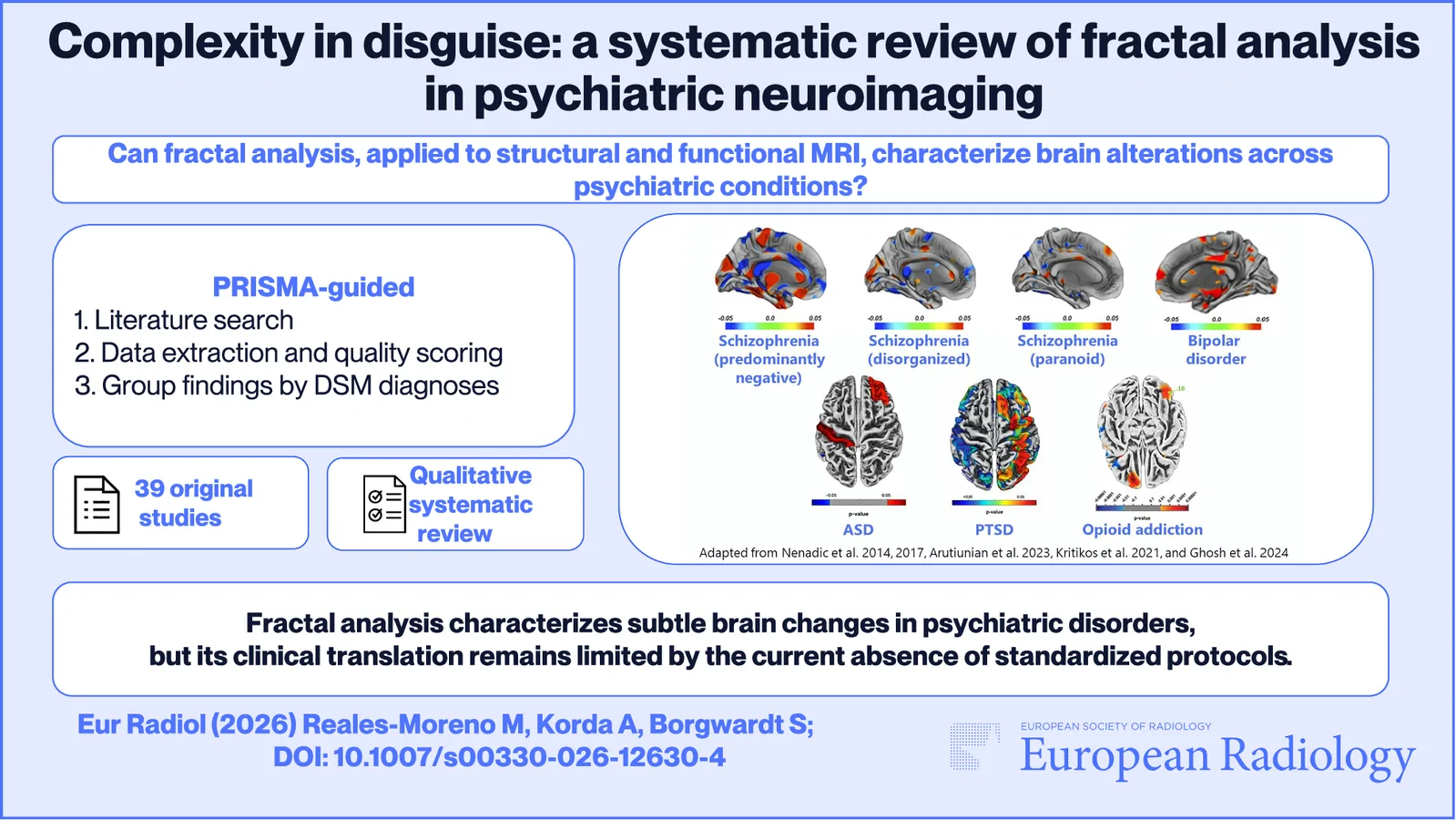

## Introduction

Readers may recall Elvis Presley’s “(You are the) devil in disguise”, a song illustrating that appearances can be misleading. Similarly, obtaining a psychiatric diagnosis is not as simple as a few clinical sessions, and conducting fractal studies involves more than applying any tool to any brain region. The title wants to suggest that fractals could provide a measure of the brain’s organization and can reveal hidden patterns in psychiatric neuroimaging data.

The World Health Organization (WHO) highlights the heterogeneity of clinical diagnoses and the global failure to provide mental health services to those in need [1]. To illustrate, only 40% of people with depression and 29% of people with psychosis receive psychiatric treatment [2]. Psychiatrists diagnose based on reported signs and symptoms, relying on classification systems like the Diagnostic and Statistical Manual of Mental Disorders (DSM-V) [3], the latest edition from 2013. In addition, psychiatrists use tests focusing on major psychiatric categories or specific conditions. These tests, influenced by the first psychometric test in the 19th century [4], remain the only essential tools despite periodic revisions over two centuries.

The human eye tends to perceive the world through Euclidean geometry, interpreting the environment in terms of dimensions: a point as zero-dimensional, a line as one-dimensional, or a plane as two-dimensional. However, this perspective cannot capture the true complexity of certain phenomena. Fractal geometry offers an analytical approach that quantifies the brain’s organization [5], revealing patterns not perceptible to the human eye. Benoit Mandelbrot played a pivotal role in unifying diverse mathematical concepts—initially casual and general work—into the field of fractals. To describe fractals in nature, Mandelbrot said that clouds are not spheres, mountains are not cones, coastlines are not circles, and bark is not smooth, nor does lightning travel in a straight line [6]. Fractals are mathematical constructs demonstrating self-similarity, non-integer dimensions, and infinite irregularity. Self-similarity refers to a system’s property where its structures remain consistent across different scales of observation, meaning that smaller parts of the system resemble the whole. Non-integer dimensions indicate that fractals occupy a space between traditional dimensions (such as lines, planes, or volumes), a description that is difficult to understand with the human eye and conventional geometry. Finally, their infinite irregularity means that no matter how closely you examine them, new patterns and details continually emerge.

Within the realm of medical research, fractal analysis holds the potential for quantifying and describing brain patterns [5, 7, 8]—offering insights into both structural and functional aspects of the brain [9]. This approach characterizes diverse brain features, including age-related changes [10–14], pathological conditions [15, 16], among others [17–21]. In psychiatric neuroimaging, fractal analysis has explored diagnostic criteria for schizophrenia [22]. These findings highlight that fractal analysis could contribute to the mental health goals established by the WHO, by enhancing the characterization and facilitation of clinical diagnoses. However, despite its growing application in characterizing psychiatric conditions, no prior attempt has systematically examined fractal patterns across multiple diagnoses. Thus, the main objective of this review was to bridge this gap by conducting a systematic analysis of fractal analysis applications in psychiatric neuroimaging—encompassing both functional and structural MRI. To achieve this aim, we followed a structured qualitative evaluation for a set of previously conducted neuroimaging studies in psychiatry, with a particular focus on the fractal analysis. The results derived from this evaluation are intended to be used to guide research directions and lay the groundwork for future clinical practice.

## Materials and methods

This study was conducted following the guidelines of the Preferred Reporting Items for Systematic Reviews and Meta-Analyses (PRISMA) 2020 [23].

### Search strategy and eligibility criteria

A systematic literature search was conducted on PubMed (Medline) to investigate the correlation between fractal results and psychiatric conditions by MRI. The search employed the following keywords: (‘autism’ OR ‘ASD’ OR ‘attention deficit hyperactivity disorder’ OR ‘ADHD’ OR ‘schizophrenia’ OR ‘psychosis’ ‘bipolar disorder’ OR ‘manic depression’ OR ‘depressive disorder’ OR ‘major depression’ OR ‘anxiety disorder’ OR ‘panic disorder’ OR ‘obsessive-compulsive disorder’ OR ‘post-traumatic stress disorder’ OR ‘substance addiction’ OR ‘substance abuse’ OR ‘eating disorder’ OR ‘anorexia’ OR ‘bulimia’ OR ‘binge eating disorder’ OR ‘personality disorder’ OR ‘borderline personality disorder’ OR ‘antisocial personality disorder’) AND (fractal) AND (‘imaging’ OR ‘neuroimaging’ OR ‘MRI’ OR ‘fMRI’ OR ‘DTI’). Other keywords were considered (‘dissociative disorder’, ‘motor disorder’, and ‘impulse-control disorder’), but there were no studies on the matter. Other databases were considered (EMBASE, Web of Science, and APA PsycINFO), but they did not contain additional publications.

Inclusion criteria encompassed fractal studies of MRI about structural and functional aspects—as well as anatomy or physiology related to psychiatric or psychological conditions in clinical assessment or diagnostic prediction using fractal analysis were included. Studies were included regardless of statistical significance, significant findings (*p* < 0.05) were specified, and studies without significant results were mentioned. All searches were restricted to articles published in English. Exclusion criteria comprised studies involving imaging tests other than MRI—e.g., electroencephalogram (EEG), positron emission tomography (PET), computed tomography (CT), retinal vascular images—visual fractal task—i.e., study without fractal analysis –, or pathology unrelated to psychiatry or psychology. Additionally, studies focusing on external factors—e.g., smoking—and their effects on psychiatric conditions were excluded. Articles failing to meet these criteria or not pertaining to a DSM-V condition were excluded from the review.

### Data extraction and quality assessment

The initial screening process for study eligibility involved data extraction based on the information provided in their abstracts. This information was organized in Microsoft Excel, which included descriptive variables such as main author, publication year, geographical region, sample size, diagnosis, age range, sex, assessment instrument, diagnostic criteria, MRI modality, fractal analysis tool, brain regions of interest, and reported outcomes. All information was verified within the full text, and any missing information was also checked in the full text and organized in the spreadsheet.

The quality of the included studies was evaluated using several established tools, including the Newcastle-Ottawa Scale (NOS) and other relevant assessment methods. For example, most of the studies were rated as high quality based on their NOS scores. Given that there were no significant differences in quality among the studies, the results were reported according to the PRISMA checklist, and the quality of the included studies was further evaluated using a multi-criteria decision-making (MCDM) approach combined with a Normalized Index Ranking Process (NIRP), as described in [24]. Four key evaluation criteria were defined: relevance to the research question (40%), methodological quality (30%), impact (20%), and innovation (10%). Raw scores were assigned according to predefined criteria [24], and studies lacking sufficient methodological detail were penalized in their score. The raw scores were then adjusted to a common scale to ensure comparability. Finally, a weighted sum of these normalized scores was calculated, allowing for a systematic ranking (between 0 and 1) of studies according to their overall contribution to the review. The entire quality evaluation procedure was strictly followed by the methodology described in [24].

Regarding bias and limitations, each study employed its own fractal analysis protocol without consensus, leading to inherent limitations. The term fractal analysis protocol refers to standardized MRI processing procedures, uniform application of fractal metrics, and consistent consideration of confounding variables across studies. Consequently, results were analyzed and described according to the particular brain regions related to the psychiatric condition studied. Any confounders were also mentioned and described as extracted information—e.g., results of autism with attention deficit disorder with hyperactivity (ADHD) comorbidity were treated as a separate condition and were not used to generalize findings to autism as a whole. Implicitly, higher quality values indicate lower risk of bias, whereas lower values indicate higher risk of bias.

### Outcome

**Fig. 1:**
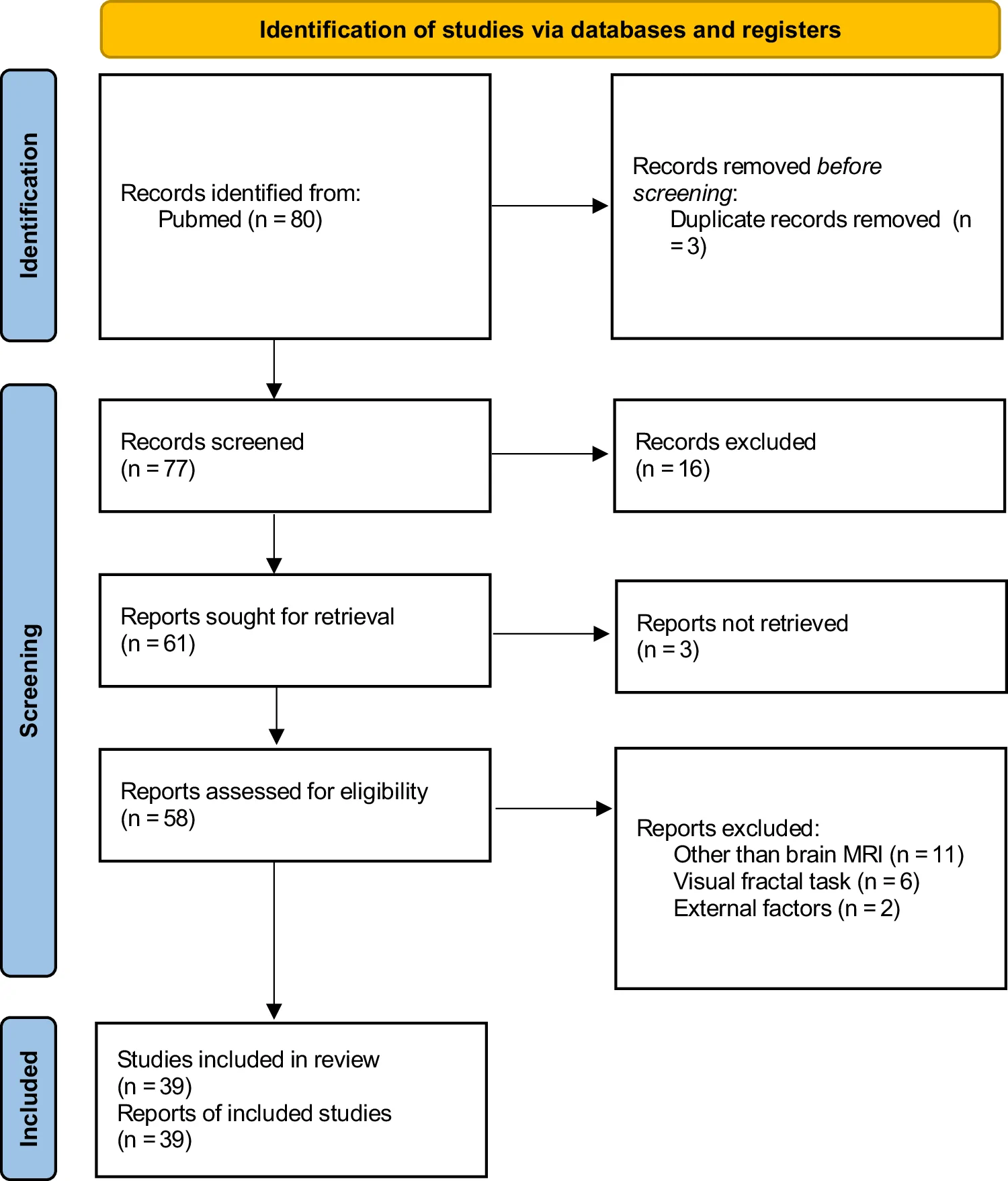
. PRISMA flow diagram. Page MJ et al [23]. https://doi.org/10.1136/bmj.n71

The process of selecting original research articles for inclusion in this review adhered to the PRISMA guidelines, as depicted in Fig. 1. Thirty-nine original studies ultimately met the predetermined inclusion criteria and were subjected to explore the potential applications of fractal analysis in psychiatry.

Following the selection of studies, they were categorized into eleven major diagnostic groups based on the nomenclature outlined in the DSM-V. These categories included:

1. Neurodevelopmental disorders
2. Schizophrenia and related disorders
3. Bipolar disorder
4. Depressive disorders
5. Anxiety disorders
6. Obsessive-compulsive disorder
7. Trauma- and stressor-related disorders
8. Feeding and eating disorders
9. Substance and addictive disorders
10. Personality disorders
11. Specifiers in psychiatry

Each study was carefully assigned to the appropriate category based on its primary focus and relevance to the respective diagnostic group. This categorization facilitated a comprehensive assessment of fractal analysis within the field of psychiatry.

## Results

For a comprehensive summary of participant demographics, diagnostic focuses, fractal metrics, and methodological nuances across studies, please refer to S1 in Supplementary Material.

### Neurodevelopmental disorders

Overall, the FD of the brain in ASD and ADHD was studied mainly in male children and adults—see Table 1.

**Table 1:** Clinical characteristics and main results of the included studies on neurodevelopmental disorders

Reference	Country	Sample and diagnosis	Age and sex (male:female ratio)	Instrument and criteria	Modality	Quality
Arutiunian et al [47]	United States and Russia	· 18 ASD · 18 Control	· 7-to-14 y/o · 13:5 and 13:5	· ADOS-2 and AQ · ICD-10	· Structural MRI (T1) · FD · ROIs	0.94
Rakshe et al [48]	India	ABIDE I database: · 47 ASD · 125 Control	· 10-to-12 y/o · 34:13 and 88:37	· ADOS and ADI-R · DSM-IV-TR	· fMRI (BOLD signal) · FD · ROIs	0.60
Khadem-Reza and Zare [49]	Iran	ABIDE II database: · 26 ASD · 26 Control	· 5-to-10 y/o · 24:2 and 25:1	· ADOS and ADI-R · ICD-9	· Structural MRI (T1) · FD (3D box-count) · ROIs	0.78
Yamagata et al [50]	Japan	· 30 ASD endophenotype · 30 Control	· 20-to-35 y/o · Male siblings by pairs	· ADOS-2, ADI-R, and AQ · DSM-IV-TR	· Structural MRI (T1) · FD · ROIs	0.68
Zhao et al [51]	China and United States	ABIDE I database: · 20 ASD · 18 Control	· 7-to-11 y/o · Only males	· ADOS and ADI-R · DSM-IV-TR	· Structural MRI (T1) · FD (3D box-count) · Cerebellar cortex	0.81
Dona et al [52]	Canada	ABIDE I database: · 55 ASD · 55 Control	· 10-to-17 y/o · 46:9 and 38:17	· ADOS and ADI-R · DSM-IV-TR	· fMRI (BOLD signal) · FD (Hurst exponent) · ROIs	0.85
Lai et al [53]	United Kingdom	· 30 ASD · 33 Control	· ≥18 y/o · Only males	· ADOS and ADI-R · DSM-IV and ICD-10	· fMRI (BOLD signal) · FD (Hurst exponent) · Whole brain and ROIs	0.87
Chen et al [54]	China and France	ABIDE II database: · 74 ASD · 40 ASD + ADHD	· 5-to-35 y/o · 66:8 and 33:7	· ADOS-G and ADI-R · ICD-9	· Structural MRI (T1) · FD · ROIs	0.76
Laatsch et al [27]	Germany and Switzerland	COMPAS trial: · 132 ADHD	· 18-to-58 y/o · 72:69	· CAARS · DSM-IV	· Structural MRI (T1) · FD · ROIs	0.65
Maier et al [26]	Germany	COMPAS trial: · 131 ADHD · 95 Control	· 18-to-48 y/o · 64:67 and 45:50	· WURS-k, CAARS, and ADHD-DC · DSM-IV	· Structural MRI (T1) · FD · ROIs	0.77
Li et al [25]	China	· 12 ADHD · 11 Control	· 11-to-15 y/o · Only males	· Health professional · DSM-IV	· Structural MRI (T1) · FD (3D box-count) · Hemispheric convolutions	0.77

*ABIDE* autism brain imaging data exchange, *ADHD* attention-deficit/hyperactivity disorder, *ADHD-DC* diagnostic checklist, *ADI-R* autism diagnostic interview-revised, *ADOS* autism diagnostic observation schedule, *AQ* autism-spectrum quotient, *ASD* autism spectrum disorder, *CAARS* Conners’ adult ADHD rating scale, *COMPAS* comparison of methylphenidate and psychotherapy in adult ADHD study, *DSM* diagnostic and statistical manual of mental disorders, *FD* fractal dimension, *ICD* international classification of diseases, *MRI* magnetic resonance imaging, *ROIs* regions of interest, *WURS-k* Wender–Utah rating scale

#### Autism spectrum disorder (ASD)

**Fig. 2:**
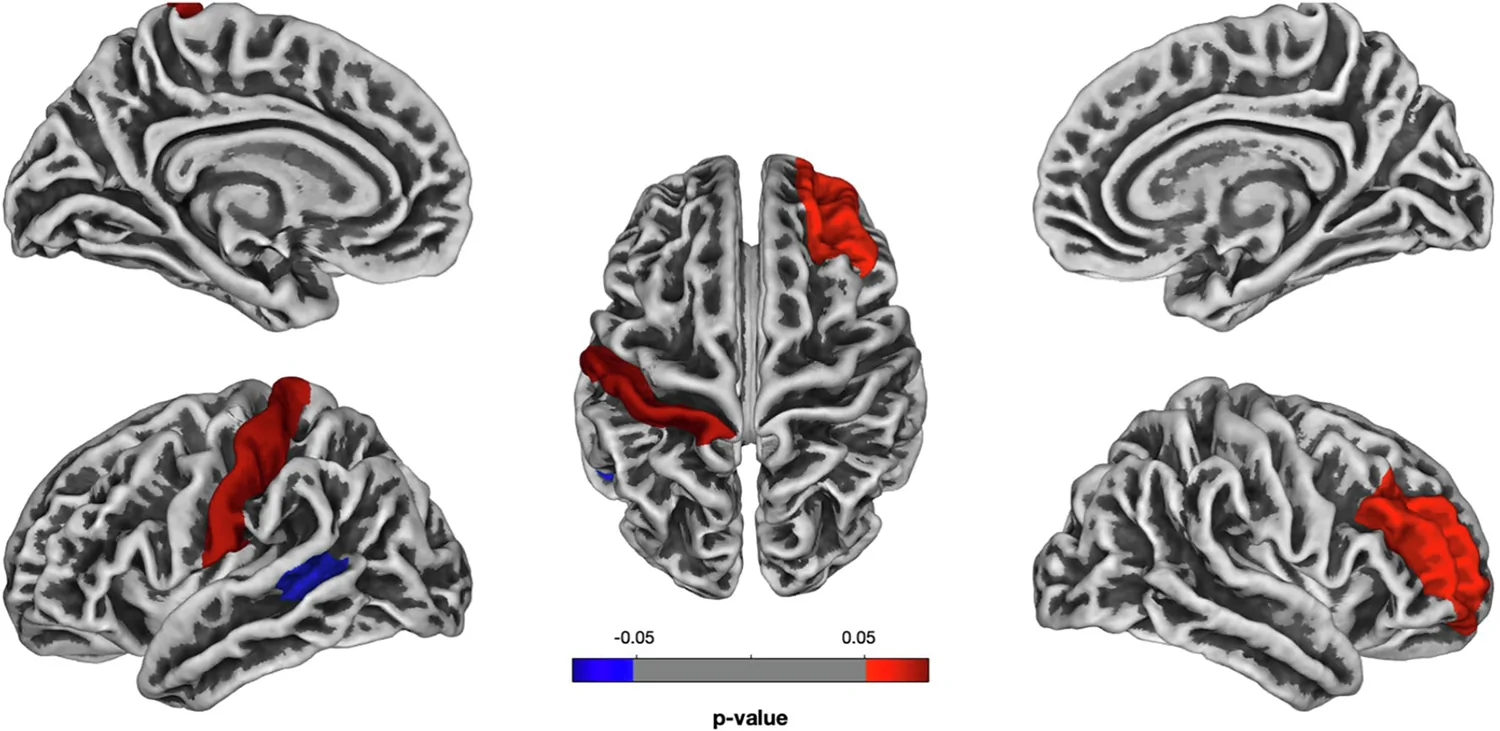
Arutiunian et al [47], showed the statistical map for the whole-brain analysis in FD (difference between ASD and control groups of children, Holm–Bonferroni corrected *p* < 0.05). Negative p-values refer to the inverse contrasts (ASD > control)

Children and adults with ASD exhibited widespread structural and functional brain alterations. Please refer to S2 in Supplementary Material for details. Lower FD and H values were observed across frontal, parietal, temporal, occipital, and limbic lobes, as well as subcortical and cerebellar regions. Notable associations emerged between reduced FD or H and symptom severity, especially in social and language domains (Fig. 2). While some patterns varied by age and comorbidities, consistent findings highlighted disrupted connectivity in frontal, parietal, and temporal areas. Network-level analyses also revealed dominant alterations within and between attention, visual, and task-control networks, underscoring large-scale connectivity disruptions in ASD.

#### Attention-deficit/hyperactivity disorder (ADHD)

Children with ADHD showed reduced FD in the left prefrontal cortex (*p* = 0.045) and altered asymmetry (*p* = 0.027), though not linked to subtypes [25]. No FD differences were found in adults [26], but inattention symptoms correlated with irregularities in paracentral and precuneus areas (*p* = 0.016) [27]—see Fig. S3 in Supplementary Material.

### Schizophrenia and related disorders

#### Schizophrenia

**Fig. 3:**
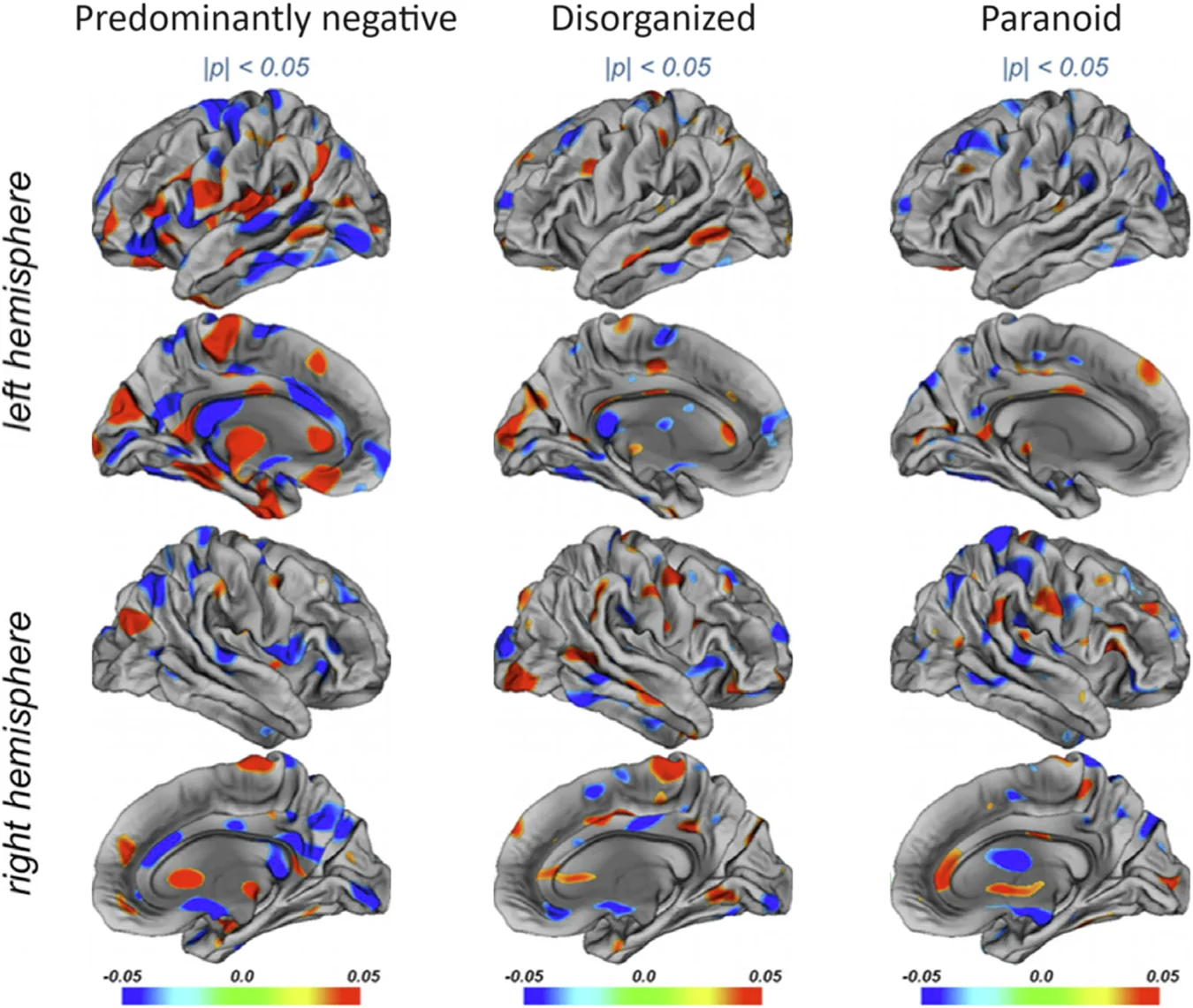
Adapted from Nenadic et al [57], in order to show the statistical significance maps comparing the FD between controls and each schizophrenia profile. Blue indicates significantly lower and red indicates significantly higher irregularity values

Structural MRI studies in adults with schizophrenia revealed widespread FD alterations, including both global and regional changes—see Table 2 and refer to S4 in Supplementary Material for details. Lower FD was observed in frontal, temporal, parietal, and cingulate cortices, with notable hemispheric asymmetries. Subcortical reductions were reported in the hippocampus and thalamus. FD patterns varied across symptom profiles, with distinct alterations in negative, disorganized, and paranoid subtypes—see Fig. 3. FD abnormalities in structural networks correlated with clinical severity. While some studies showed increased FD in gray-white boundaries, most reported global and regional reductions, supporting less structural irregularity in schizophrenia.

**Table 2:** Clinical characteristics and main results of the included studies on schizophrenia and related disorders

Reference	Country	Sample and diagnosis	Age and sex (male:female ratio)	Instrument and criteria	Modality	Quality
Fan et al [55]	China	· 203 First-episode schizophrenia · 131 Control	· 19-to-34 y/o · 95:108 and 73:58	· PANSS and MCCB · DSM-IV	· Structural MRI (T1) · FD (SPH) · ROIs (connections, morphological similarity)	0.90
Zhao et al [56]	China and United States	· 19 Schizophrenia · 19 Control	· 26-to-50 y/o · 17:2 and 13:6	· Health professional · DSM-IV	· Structural MRI (T1) · FD (3D box-count) · Brainstem and subcortical structures	0.81
Squarcina et al^a^ [29]	Italy	· 25 Schizophrenia · 11 Bipolar · 39 Control	· 23-to-50 y/o · 15:10, 5:6, and 17:22	· Health professional · DSM-IV	· Structural MRI (T1) · FD (2D box-count) · Whole-brain and lobes	0.79
Nenadic et al [57]	Germany	· 87 Schizophrenia · 108 Control	· 22-to-46 y/o · 48:39 and 68:40	· SANS and SAPS · DSM-IV and DSM-IIIR	· Structural MRI (T1) · FD (SPH) · Whole brain and ROIs	0.81
Yotter et al [58]	Germany and USA	· 87 Schizophrenia · 108 Control	· 22-to-47 y/o · 48:39 and 68:40	· SANS and SAPS · DSM-IV	· Structural MRI (T1) · FD (box-count and SPH) · Whole brain and ROIs	0.86
Yotter et al [59]	Germany and USA	· 87 Schizophrenia · 108 Control	· 22-to-47 y/o · 48:39 and 68:40	· SANS and SAPS · DSM-IV	· Structural MRI (T1) · FD (SPH) · Hemispheres and lobes	0.73
Sandu et al [60]	Norway	· 7 Schizophrenia · 6 Control	· 19-to-51 y/o · Males and females	· BPRS · DSM-IV	· Structural MRI (T1) · FD (box-count and Minkowski-Bouligand) · Hemispheres and whole brain	0.76
Ha et al^a^ [34]	Korea	· 45 Schizophrenia · 50 Obsessive-compulsive disorder · 26 Control	· 19-to-34 y/o · 26:19, 34:16, and 16:10	· PANSS and Y-BOCS · DSM-IV	· Structural MRI (T1) · FD (3D box-count) · Hemispheres and whole brain	0.78
Narr et al [61]	United States	· 50 First-episode schizophrenia · 50 Control	· 21-to-35 y/o · 33:17 and 33:17	· SADS-C and SANS · DSM-IV	· Structural MRI · FD (3D) · Hemispheres by lobes	0.81
Bullmore et al 1994^a^ [28]	United Kingdom	· 39 Schizophrenia · 23 Manic-depressive · 31 Control	· <50 y/o · 56:37 (total)	· Health professional · DSM-III-R	· Structural MRI (STIR) · FD (2D box-count) · Whole brain	0.91

*BPRS* brief psychiatric rating scale, *DSM* diagnostic and statistical manual of mental disorders, *FD* fractal dimension, *MCCB* matrics consensus cognitive battery, *MRI* magnetic resonance imaging, *PANSS* positive and negative syndrome scale, *ROIs* regions of interest, *SADS-C* schedule for affective disorders and schizophrenia, *SAPS* scales for the assessment of positive symptoms, *SANS* scale for the assessment of negative symptoms, *SPH* spherical harmonics-based method, *Y–BOCS* Yale–Brown obsessive-compulsive scale

^a^ Study that included different diagnostic groups

### Bipolar disorder

**Fig. 4:**
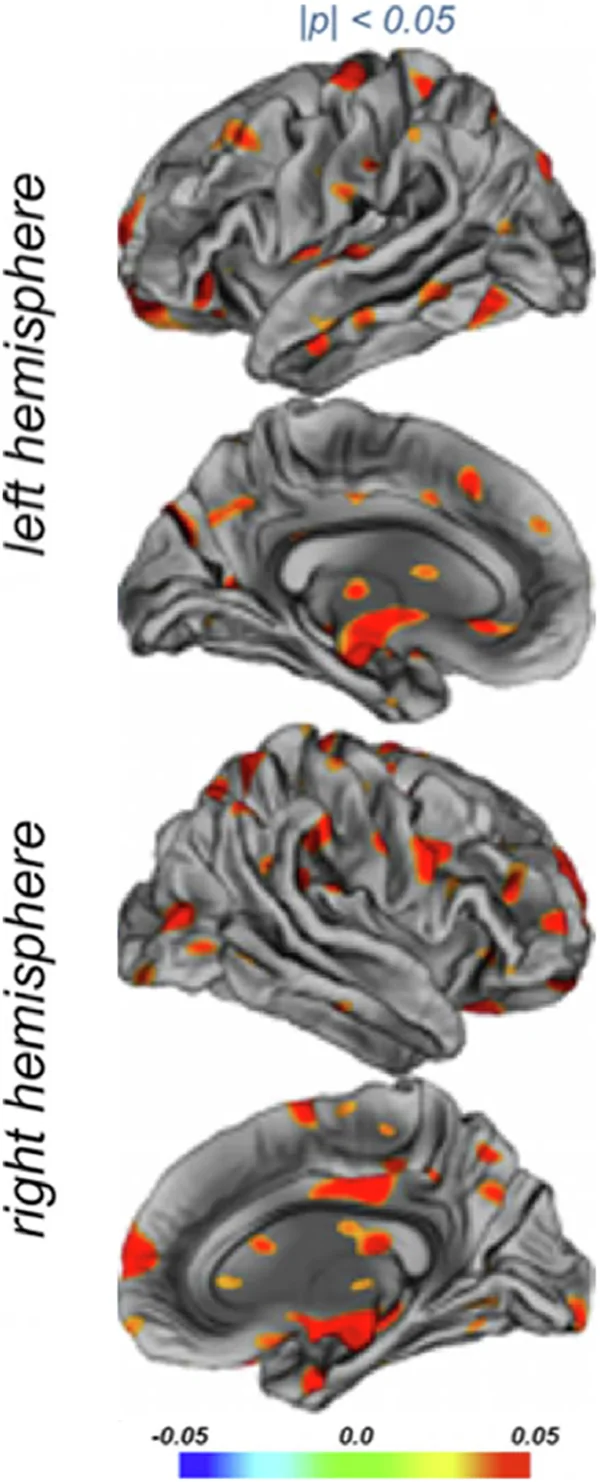
Adapted from Nenadic et al [30], in order to show the statistical significance maps comparing the FD between controls and bipolar disorder. Blue indicates significantly lower, and red indicates significantly higher irregularity values

Bipolar disorder was studied using structural MRI in adults—see Table 3. Controlling for effects of brain size, bipolar disorder showed increased global FD (*p* < 0.05) [28], but also regionally reduced FD in frontal gray matter (*p* = 0.05) [29]. Regional patterns—see Fig. 4—included increased FD in left lateral orbitofrontal cortex and right precuneus, but decreased FD in right caudate, middle frontal, right pars orbitalis, left fusiform, entorhinal, and posterior cingulate cortices [30].

**Table 3:** Clinical characteristics and main results of the included studies on bipolar, depressive, and anxiety disorders

Reference	Country	Sample and diagnosis	Age and sex (male:female ratio)	Instrument and criteria	Modality	Quality
Guo et al [31]	China and Australia	· 20 Bipolar disorder (type I) · 17 Control	· 10-to-18 y/o · 12:8 and 7:10	· YMRS and MFQ · DSM-IV	· fMRI (BOLD signal) · FD (Hurst Exponent) · ROIs	0.83
Nenadic et al [30]	Germany and United States	· 18 Bipolar disorder (*n* = 15 type I, *n* = 3 type II) · 26 Control	· 25-to-50 y/o · 7:11 and 15:11	· YMRS and HAM-D · DSM-IV-R and DSM-V	· Structural MRI (T1) · FD (SPH) · ROIs	0.79
Squarcina et al^a^ [29]	Italy	· 25 Schizophrenia · 11 Bipolar · 39 Control	· 23-to-50 y/o · 15:10, 5:6, and 17:22	· Health professional · DSM-IV	· Structural MRI (T1) · FD (2D box-count) · Whole brain and lobes	0.79
Bullmore et al^a^ [28]	United Kingdom	· 39 Schizophrenia · 23 Manic-depressive · 31 Control	· <50 y/o · 56:37 (total)	· Health professional · DSM-III-R	· Structural MRI (STIR) · FD (2D box-count) · Whole brain	0.91
Pang et al [32]	China	· 44 Non-suicidal self-injury (related to depressive disorder) · 44 Control	· 15-to-18 y/o · 9:35 and 16:28	· HAM-D, CRSNSSI, ANSAQ, and BPAQ	· Structural MRI · FD · ROIs	0.56
Gentili et al [62]	Italy, Romania, and the United States	· 36 Healthy adults assessed for social anxiety disorder	· 23-to-31 y/o · 20:16	· LSAS and BFNE · DSM-IV-TR	· Functional MRI · FD (Hurst exponent) · ROIs	0.55
Mujica-Parodi et al [33]	United States	· 22 Healthy adults (skydiving) · 30 Healthy adults (control)	· 18-to-48 y/o · 20:2 and 18:12	· NEO PI-R, PSS, ARQ, STAI, and SSS	· Functional and structural MRI · Fractal irregularity (PSSI) · ROIs	0.57
Olejarczyk [63]	Poland	· 65 Healthy adults in a state of increased anxiety	N/A	N/A	· fMRI (BOLD signal) · FD (1D-Higuchi) · ROIs	0.50

*ANSAQ* adolescent non- suicidal self-injury assessment questionnaire, *ARQ* attitude to risk questionnaire, *BFNE* brief fear of negative evaluation scale, *BPAQ* Buss–Perry aggression questionnaire, *CRSNSSI* clinician-rated severity of non- suicidal self-injury, *DSM* diagnostic and statistical manual of mental disorders, *FD* fractal dimension, *HAM-D* Hamilton depression rating scale, *LSAS* Liebowitz social anxiety scale, *MFQ* mood and feelings questionnaire, *MRI* magnetic resonance imaging, *NEO PI* neuroticism, extraversion, openness personality inventory, *PSS* perceived stress scale, *PSSI* power spectrum scale invariance, *ROIs* regions of interest, *SPH* spherical harmonics-based method, *SSS* somatic symptom scale, *STAI* state-trait anxiety inventory, *YMRS* Young Mania rating scale

^a^ Study that included different diagnostic groups

Functionally, bipolar I subjects exhibited higher H values during response inhibition—Go-Nogo—tasks in left (*p* = 0.025) superior frontal gyrus (BA 10), left (*p* = 0.025) inferior frontal gyrus (BA 45), right (*p* = 0.041) orbital gyrus (BA 47), left (*p* = 0.021) precentral gyrus (BA 6), left (*p* = 0.018) inferior temporal gyrus (BA 20), left (*p* = 0.018) and right (*p* = 0.025) inferior parietal lobule (BA 39), and right (*p* = 0.027) insular gyrus (BA 48)—indicating reduced flexibility in adaptive control [31].

### Depressive disorders

Despite depression’s global prevalence, fractal analysis remains limited. Pang et al [32] was the only known study using this approach to assess non-suicidal self-injury in adolescents—often linked to major depressive disorder—and found no significant FD differences between affected and control groups—see Table 3.

### Anxiety disorders

#### Anxiety state (healthy adults)

Fractal characteristics of anxiety were examined in healthy adults using primarily functional MRI—see Table 3.

Gentili et al 2015 found positive correlations between H values and clinical scores: BFNE scores—Brief Fear of Negative Evaluation Scale—correlated with the precuneus and posterior cingulate (i.e., the higher the fear of negative evaluation is, the higher the irregularity is), while LSAS scores—Liebowitz Social Anxiety Scale–correlated (*p* < 0.01) with the precuneus, inferior parietal sulcus, and right parahippocampus.

Studies by Olejarczyk 2007 and Mujica-Parodi et al [33] showed positive correlations between fractality and regulatory control in the left amygdala and left inferior frontal gyrus (BA 45), key areas in the prefrontal-limbic circuit involved in emotional regulation and stress response. Olejarczyk also highlighted the hippocampus and dorsolateral prefrontal cortex (BA 9).

### Obsessive-compulsive disorder (OCD)

Ha et al [34] reported that FD-based logistic regression correctly classified 88% of OCD cases—see Table 4. OCD was associated with significantly lower FD in the whole brain, both hemispheres, gray matter, and intracranial volume.

**Table 4:** Clinical characteristics and main results of the included studies on obsessive-compulsive, trauma- and stressor-related, and feeding and eating disorders

Reference	Country	Sample and diagnosis	Age and sex (male:female ratio)	Instrument and criteria	Modality	Quality
Ha et al^a^ [34]	Korea	· 45 Schizophrenia · 50 OCD · 26 Control	· 19-to-34 y/o · 26:19, 34:16, and 16:10	· PANSS and Y-BOCS · DSM-IV	· Structural MRI (T1) · FD (3D box-count) · Hemispheres and whole brain	0.78
Kritikos et al [35]	United States	World Trade Center responders: · 47 PTSD · 52 without PTSD	· 44-to-65 y/o · 78.8% males	· Health professional · DSM-IV	· Structural MRI (T1) · FD (3D) · Whole brain and ROIs	0.70
Vatheuer et al^a^ [36]	Germany and the Netherlands	· 18 BPD + PTSD · 39 BPD · 42 Control	· 20-to-43 y/o · Only females	· BPDSI, BPD checklist, BSI, and ITEC · DSM-IV	· Structural MRI (T1) · FD · Whole brain and ROIs	0.88
Collantoni et al [40]	Italy	· 38 Anorexia nervosa · 20 Recovered from anorexia nervosa · 38 Control	· 19-to-33 y/o · Only females	· EDI · DSM-V	· Structural MRI (T1) · FD · Thalamus	0.81
Collantoni et al [37]	Italy	· 58 Anorexia nervosa (38 acute and 20 recovered) · 38 Control	· 15-to-40 y/o · Only females	· Health professional · DSM-V	· Structural MRI (T1) · FD (3D box-count) · Whole brain and ROIs	0.82
Cascino et al [39]	Italy	· 22 Anorexia nervosa · 10 Recovered from anorexia nervosa · 24 Bulimia nervosa · 35 Control	· 18-to-39 y/o · Only females	· EDI · DSM-V	· Structural MRI (T1) · FD · Brain lobes	0.85
Nickel et al [38]	Germany	· 34 Anorexia nervosa · 24 Recovered from anorexia nervosa · 41 Control	· 20-to34 y/o · Only females	· EDE and EDI-2 · DSM-IV	· Structural MRI (T1) · FD · ROIs	0.76

*BPD* borderline personality disorder, *BPDSI* borderline personality disorder severity index, *BSI* brief symptom inventory, *DSM* diagnostic and statistical manual of mental disorders, *EDE* eating disorder examination, *EDI* eating disorder inventory, *FD* fractal dimension, *ITEC* interview for traumatic events in childhood, *MRI* magnetic resonance imaging, *PANSS* positive and negative syndrome scale, *PTSD* post-traumatic stress disorder, *ROIs* regions of interest, *Y–BOCS* Yale–Brown obsessive-compulsive scale

^a^ Study that included different diagnostic groups

### Trauma- and stressor-related disorders

#### Post-traumatic stress disorder (PTSD)

Fractal patterns in PTSD were studied using functional MRI in adults—see Table 4.

**Fig. 5:**
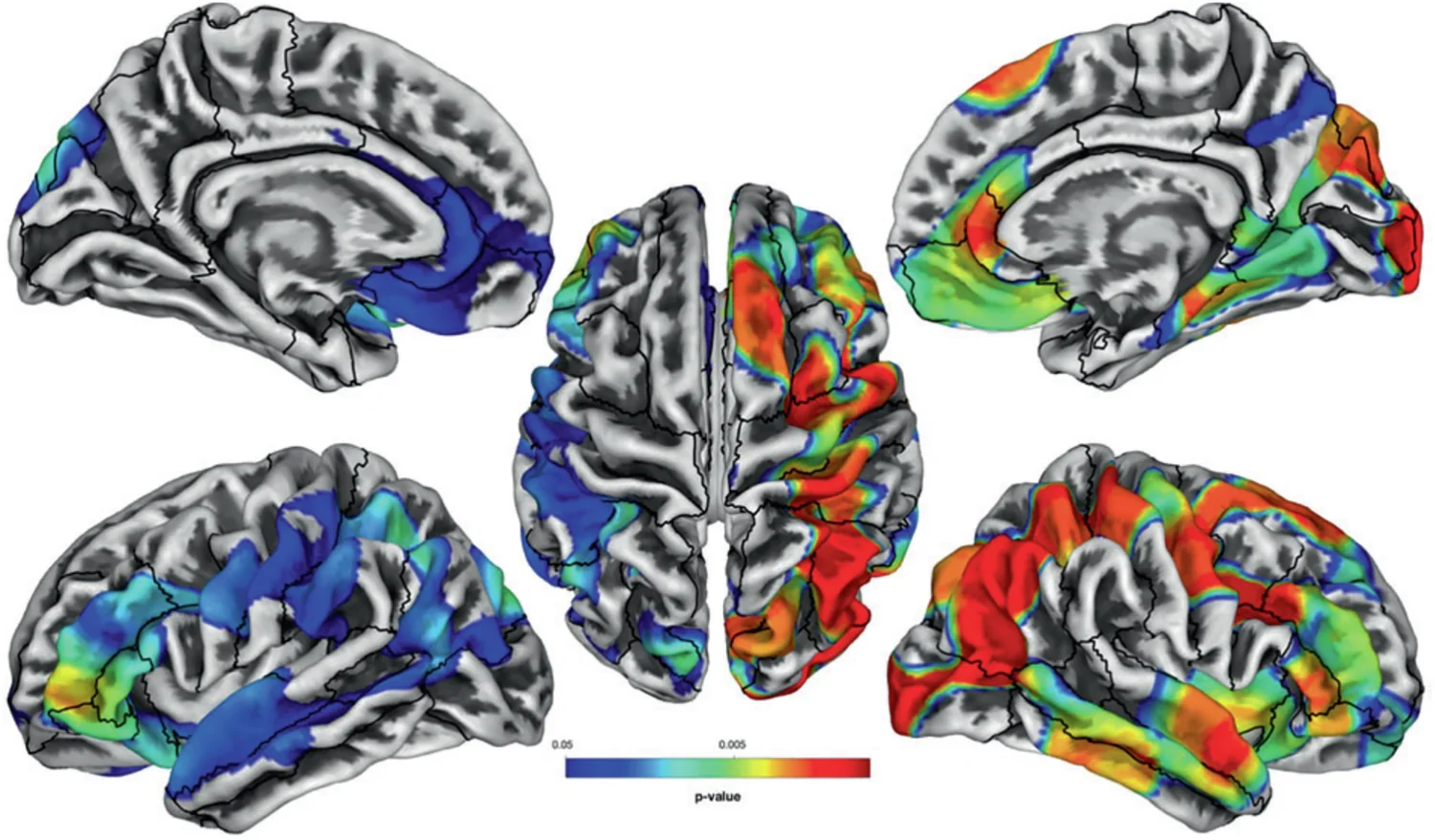
Kritikos et al [35] showed the analyses of FD using surface-based morphometry comparing PTSD negative to PTSD positive responders. Regions lacking significant differences in FD between groups are shown in gray, while statistically significant results are shown using a rainbow color map ranging from blue to red

Kritikos et al [35]—focusing on males—found bilateral and unilateral FD reductions in frontal, parietal, and temporal cortices in World Trade Center-related PTSD. Greater symptom severity—including reexperiencing, avoidance, hyperarousal, and negative affect—correlated with stronger FD changes—see Fig. 5.

In females, Vatheuer et al [36] observed that BPD patients with comorbid PTSD showed similar brain patterns to those without PTSD, particularly in the hippocampus, amygdala, and anterior cingulate cortex (ACC)—suggesting shared structural features linked to stress and emotion regulation.

### Feeding and eating disorders

#### Anorexia nervosa and bulimia nervosa

Fractal analysis of anorexia and bulimia nervosa has focused on adult females using structural MRI, with limited representation of adolescence—see Table 4.

Globally, Collantoni et al [37] found lower mean cortical FD in acute anorexia nervosa compared to controls (*p* < 0.001), while recovered individuals showed mixed FD alterations across different regions—not specified.

Regionally, Nickel et al [38] reported increased FD in the left precentral gyrus in acute anorexia, but no differences in recovered individuals. Collantoni et al [37] observed lower FD in parietal regions in both acute and recovered groups. Cascino et al [39] found no significant FD differences across acute anorexia, recovered anorexia, bulimia nervosa, and controls.

Subcortically, Collantoni et al [40] reported reduced FD in the whole thalamus and some nuclei—not specified—in acute anorexia. The recovered group showed altered FD in the right laterodorsal and central lateral nuclei.

### Substance and addictive disorders

#### Drug users

Only male adults were assessed for opioid, cocaine, or methamphetamine use—see Table 5.

**Table 5:** Clinical characteristics and main results of the included studies on substance and addictive, and personality disorders, and psychiatric specifiers

Reference	Country	Sample and diagnosis	Age and sex (male:female ratio)	Instrument and criteria	Modality	Quality
Ghosh et al [41]	India	· 69 Opioid use disorder (52 heroin and 17 non-heroin) · 25 Control	· 31-to-32 y/o · Only males	· MINI 6.0 · ICD-10	· Structural MRI (T1) · FD · ROIs	0.80
Trevisan et al [42]	Italy	· 52 Chronic cocaine users · 36 Control	· 22-to-38 y/o · Only males	· BIS-11 · DSM-IV	· Structural MRI (T1) · FD (SPH) · ROIs	0.81
Siyah Mansoory et al [43]	Iran	· 17 Methamphetamine drug users · 18 Control	· 22-to-46 y/o · Only males	N/A	· fMRI (BOLD signal) · FD (3D box-count) · ROIs	0.75
Varley et al [64]	United Kingdom and United States	· 20 LSD volunteers · 15 Psilocybin volunteers	· ≥21 y/o · 16:4 and 10:5	N/A	· fMRI (BOLD signal) · FD (1D-Higuchi) · ROIs (neural networks)	0.62
Weber et al [44]	Canada	· 11 Alcohol volunteers (Intoxicated and Non-intoxicated states)	· 22-to-38 y/o · Only males	N/A	· fMRI (BOLD signal) · FD (Hurst exponent) · Default mode network	0.64
Vatheuer et al^a^ [36]	Germany and the Netherlands	· 18 BPD with PTSD · 39 BPD · 42 Control	· 20-to-43 y/o · Only females	· BPDSI, BPD checklist, BSI, and ITEC · DSM-IV	· Structural MRI (T1) · FD · Whole brain and ROIs	0.88
Parekh et al [45]	India	· 15 Acute retarded catatonic state · 15 Control	· 18-to-41 y/o · 7:8 and 7:8	· BFCRS and NCRS · DSM-V	· fMRI (BOLD signal) · Cortical surface fractality · ROIs (neural networks)	0.66

*BFCRS* Bush–Francis Catatonia Rating Scale, *BIS* behavioral inhibition scale, *BPD* borderline personality disorder, *BPDSI* borderline personality disorder severity index, *BSI* brief symptom inventory, *DSM* diagnostic and statistical manual of mental disorders, *FD* fractal dimension, *ICD* international classification of diseases, *ITEC* interview for traumatic events in childhood, *LSD* lysergic acid diethylamide, *MINI* mini international neuropsychiatric interview, *MRI* magnetic resonance imaging, *NCRS* Northoff Catatonia rating scale, *ROIs* regions of interest, *SPH* spherical harmonics-based method

**Fig. 6:**
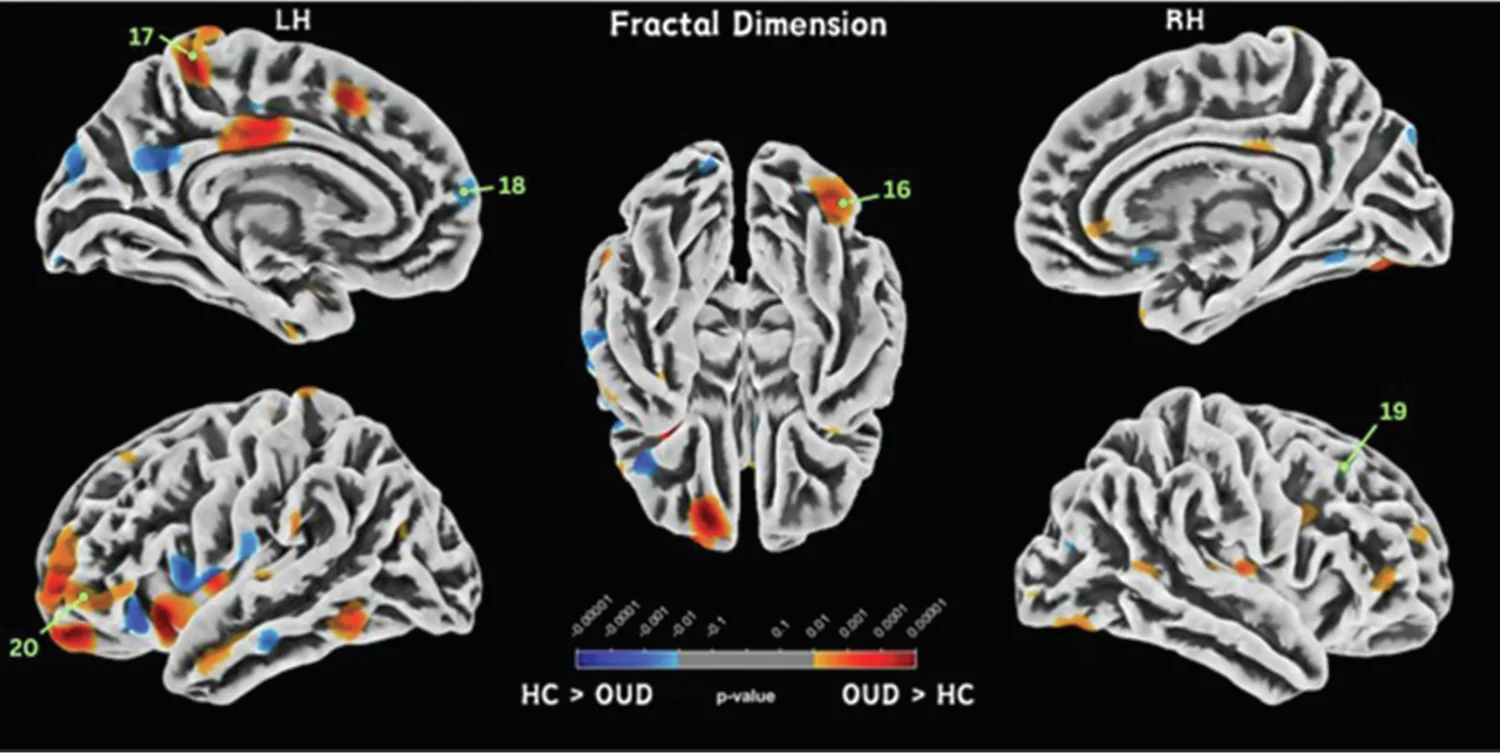
Ghosh et al [41] presented brain regions with significant statistical differences between men with opioid use disorder and controls (*p* < 0.01). The color bar indicates regions significantly higher (red) or lower (blue) in FD

In Ghosh et al [41], opioid users—primarily heroin users—exhibited higher FD in the right inferior occipital gyrus and sulcus (*p* = 0.005), left paracentral gyrus and sulcus (*p* = 0.002), left transverse frontopolar gyrus and sulcus (*p* = 0.006), right middle frontal gyrus (*p* = 0.001), and left lateral orbital sulcus (*p* = 0.001)—see Fig. 6.

Trevisan et al [42] found that cocaine users had reduced cortical folding irregularity (CCF)—determined using FD—in the left insula (*p* = 0.008), left supramarginal gyrus (*p* = 0.008), and left medial orbitofrontal cortex (*p* = 0.039). In the medial orbitofrontal cortex, higher CCF was correlated with later cocaine use onset (*p* = 0.028), while lower CCF was correlated with higher attentional impulsivity of the BIS score (*p* = 0.048).

Siyah Mansoory et al [43] observed that methamphetamine users had reduced FD in global efficiency and clustering coefficient, alongside increased characteristic path length, indicating reduced brain network efficiency and organization—i.e., less efficient communication across the brain network.

#### Induced intoxication (volunteers)

Male adults participated in studies of induced intoxications using functional MRI—see Table 5. These studies focus on acute brain activity changes, not addiction.

In Varley et al 2020, LSD (Lysergic acid diethylamide) volunteers showed increased FD in the fronto-parietal (*p* = 0.001), dorsal attention (*p* < 0.001), and visual (*p* = 0.001) networks—linked to attention, perception, and sensory processing. In the same study, psilocybin volunteers had increased FD in the dorsal attention network (*p* = 0.05), suggesting a focus on attentional processes.

In Weber et al [44]—on Default Mode Network (DMN)— the ethanol induced peak in the right basal ganglia, at 60 min post-consumption, increased H values (*p* < 0.01) in the right cerebellum, culmen, right superior temporal gyrus, substantia nigra, left lingual gyrus, right insula, left cuneus, left putamen, left posterior cingulate cortex, right caudate, and thalamus.

### Personality disorders

#### Borderline personality disorder (BPD)

In Vatheuer et al 2021, adult women with BPD showed higher FD in the right superior parietal cortex compared to controls—see Table 5—potentially linked to cognitive and emotional processing differences.

### Specifiers in psychiatry

#### Catatonic state

Parekh et al [45] assessed catatonia in young adults using structural and functional MRI—see Table 5. Network analysis revealed significant differences in the cerebellar, salience, default mode, and sensorimotor networks. Patterns of increased and decreased fractality were found across sensorimotor, frontal, parietal, temporal, and cerebellar regions. Catatonia was also associated with reduced cortical surface fractality in the right insular cortex. Increased brain activity was observed mainly in the left cerebellum, while decreased activity spanned various cortical regions.

Treatment response analysis showed that lorazepam responders had higher fractality in ROI-to-ROI connectivity in frontal, occipital, and left paracingulate regions compared to non-responders, indicating different brain connectivity profiles linked to treatment outcomes.

## Discussion

The reviewed studies emphasize the utility of fractal measures—primarily FD and the Hurst Exponent (H)—in characterizing psychiatric conditions. These metrics allow for an analysis of brain fractality, offering insights into brain organization across psychiatric populations. Although each published study to date employs its own specific pipeline, most workflows share a set of common conceptual stages: MRI acquisition, image preprocessing, adjustment to the selected fractal metric, extraction of structural or functional features, statistical comparison between groups, and clinical or cognitive interpretation.

Geographic distribution plays an important role in shaping psychiatric diagnostic definitions and epidemiological trends. The United States leads in contributing to the literature, followed by Germany and Italy. This distribution reflects both the accessibility of advanced neuroimaging techniques and the epidemiological emphasis on psychiatric conditions. However, differences in diagnostic definitions across professionals and countries may introduce variability in findings, emphasizing the role of cultural influences on psychiatry—and therefore in psychiatric neuroimaging. While many studies considered covariates like handedness, IQ, or age, this is not a general practice. The use of demographic covariates is a crucial consideration that could demonstrate more significant differences [28].

### Fractal analysis of the psychiatric neuroimaging

For an in-depth overview of fractal neuroimaging findings across diverse psychiatric diagnoses, including region-specific alterations and clinical correlations, please refer to S5 in Supplementary Material.

As seen in Table S6 (Supplementary Material), the frontal and parietal lobes—followed by the limbic lobe—are the most prominently affected across a wide range of conditions. Among the affected ROIs, in order of frequency, there were the cingulate, orbitofrontal, and prefrontal cortices—to a lesser extent, the precuneus, parietal cortex, superior parietal lobule, fusiform gyrus, and insular cortex.

### Implications and future directions

Many brain structures—such as the cortex and neural networks—exhibit fractal geometry, with the FD obtained through the box-counting method being the most widely studied metric [5]. Fractal analysis has been applied in psychiatric research, revealing imaging alterations compared to controls, although it has not yet been incorporated into diagnostic or therapeutic frameworks. The main limitation lies in the absence of standardized protocols for image processing and fractal calculation. To validate its use, longitudinal studies are needed to track fractal changes in disorders and treatment—i.e., comparing treated vs untreated patients, as well as pre- and post-treatment effects. Future studies should also adopt a translational perspective, linking fractal measures to clinical symptoms, treatment response, age, and other covariates, to better understand how individual variability influences FD. Underrepresented conditions, such as depression and anxiety, deserve more attention.

Due to methodological heterogeneity, conducting a meta-analysis was not feasible; however, greater standardization could make this possible and yield more robust results in psychiatry.

Computational tools—such as machine learning and artificial intelligence—may also improve diagnostic classification. Importantly, rather than expecting a biomarker for a disorder, it is more likely to identify distinct brain patterns associated with clusters of symptoms. Finally, the consistent alterations detected across multiple psychiatric conditions in the frontal lobe organization by fractal analyses confirm a major role of these structures as a hub in psychiatric pathophysiology [9].

## Conclusions

Since approximately 75% of psychiatric disorders manifest before the age of 25 [46], it is necessary to characterize neurodevelopment and implement early interventions in clinical practice. In this context, fractal techniques are particularly valuable, as they can provide complementary information to conventional MRI, which to date has not succeeded in establishing a clinical application despite the extensive existing literature.

## Supplementary information

ELECTRONIC SUPPLEMENTARY MATERIAL

## Abbreviations

ADHD: Attention deficit disorder with hyperactivity
ASD: Autism spectrum disorder
BPD: Borderline personality disorder
DSM: Diagnostic and statistical manual of mental disorders
ICD: International classification of diseases
LSD: Lysergic acid diethylamid
MRI: Magnetic resonance imaging
PTSD: Post-traumatic stress disorders
ROI: Region of interest
WHO: World Health Organization
