# Supplementary material for: Complexity in disguise: a systematic review of fractal analysis in psychiatric neuroimaging
Source: Eur Radiol. 2026 May 26;36(10):7760–75. doi: 10.1007/s00330-026-12630-4 (PMC13574870; doi:10.1007/s00330-026-12630-4)
Supplement: Supplementary file 1 [file 330_2026_12630_MOESM1_ESM.pdf]

ELECTRONIC SUPPLEMENTARY MATERIAL

Complexity in disguise: A systematic review of fractal analysis in psychiatric neuroimaging

S1. Summary of participant demographics, diagnostic focuses, fractal metrics, and methodological nuances across studies

The results reveal a predominant focus on young adult populations with psychiatric conditions, often compared with control groups. While our objective was not specifically centered on covariates, it is crucial to consider the demographic information present in each study [9-13, 16-20] – e.g., right-handed individuals, intelligence quotient, sex, age, and geographical location –, so some of the selected studies managed this situation by matching participants on covariates. The geographical distribution of fractal studies is an important aspect to consider, as countries play a significant role in shaping diagnostic definitions and epidemiological trends in psychiatry. Thus, the United States contributed significantly to the knowledge gap of fractal neuroimaging in psychiatry, followed by Germany and Italy.

Among the major diagnostic categories outlined in the DSM-V, "schizophrenia and related disorders" have garnered considerable attention, comprising approximately 24% of the studies reviewed. Additionally, "Autism Spectrum Disorder" emerges as another prominent focus of investigation.

Notably, the evaluation indicates that the fractal dimension (FD) is the most employed analysis method. The FD reflects the organization of brain structures, with higher values indicating greater irregularity and lower values suggesting reduced irregularity. It is essential to note that FD can be conducted in two- or three-dimensional space. In addition to FD, the Hurst exponent (H) is another crucial metric utilized in fractal analysis. While FD focuses on how space is filled – the 'shape' of the fractal –, while the H examines the statistical properties and self-similarity as the scale of observation changes. It is pertinent to consider the implications of whole-brain FD calculations, as these may potentially underestimate local abnormalities due to the inclusion of a high percentage of brain tissue, thereby potentially masking important findings.

Often, studies refer to the term "complexity" when referring to fractal analysis. However, it is important to note that the proper terminology should have been used, as complexity and fractality have different meanings – as explained in Korda et al. 2023 – another nod to the title. Fractality describes geometric patterns and how a structure occupies space, while complexity reflects the dynamic and multifactorial interactions that characterize a system's behavior. In other words, fractals quantify how irregular a system is, but this measure is not synonymous with the degree of complexity.

Moreover, most studies focus on the anatomical description of brain areas, while others do so through Brodmann Areas (BA) – in other words, focused on the arrangement of the soma of neurons in the cerebral cortex. Our review reveals a higher frequency of frontal lobe findings among fractal studies.

S2. Autism Spectrum Disorder (ASD)

In terms of global measures, adults with ASD had global H values significantly lower on the gray matter ($p = 0.011$) and the white matter ($p = 0.031$), but there were not significant differences on the total brain volume [30].

Regarding symptomatology, Arutiunian et al. 2023 in children with ASD, the structural ROIs defined by FD – i.e., right rostral middle frontal gyrus, left postcentral gyrus, and left superior temporal sulcus – were not associated with the severity of autism, while lower FD in the temporal cortex was associated with severe language impairment ($p = 0.02$) and higher non-verbal IQ ($p = 0.01$). In terms of functional connectivity, children with autism demonstrated that the ADI social interaction score was positively correlated with the left BA 23 ($p = 0.03$) and right uvula of the vermis ($p = 0.03$), the right amygdala was positively correlated with the ADI verbal communication score ($p = 0.02$) and ADI total score ($p = 0.05$), but there was not significant correlations with ADOS scores [29]; while in adults the severity of ASD symptoms was negatively related with H values in the retrosplenial and right anterior insular cortices [30]. Besides, in children and adults with autism and comorbid ADHD, the FD was negatively correlated with the ADI-R social score [31].

The frontal lobe highlighted many significant differences compared with typical developing. Children with ASD showed lower FD in the structure of the right rostral middle frontal gyrus ($p = 0.03$) [24] and ($p = 0.014$) [26], compared to Khadem-Reza & Zare, 2022 that also indicated decreased FD in the right caudate middle frontal gyrus ($p = 0.001$), right frontal pole ($p = 0.006$), right superior frontal gyrus (p=0.003), right lateral orbitofrontal cortex ($p = 0.002$), left ($p = 0.022$) and right ($p = 0.003$) medial orbitofrontal cortex, right precentral gyrus ($p = 0.002$), right paracentral lobule ($p = 0.004$), right pars opercularis ($p = 0.005$), left ($p = 0.011$) and right ($p = 0.008$) pars triangularis, and left ($p = 0.034$) and right ($p = 0.004$) pars orbitalis. Yamagata et al. 2019, in adults with ASD endophenotype[1], revealed that the mean FD in the right medial orbitofrontal gyrus and right caudal middle frontal gyrus were negatively correlated with AQ attention to detail score ($p = 0.017$ and $p = 0.039$, respectively). In terms

[1] The endophenotype is a measurable and biological characteristic that reflects the genetic liability for a clinical condition and exists between genes and clinical phenotypes in both affected individuals and their unaffected relatives.

of functional connectivity, youth with ASD showed lower H values in the left BA 33 ($p = 0.03$) [29] – in contrast to Lai et al., 2010, since adults with autism showed lower H values in the medial prefrontal cortex (BA 10), supplementary motor area (BA 6 and 8), and inferior frontal gyrus (BA 44 and 45).

In the parietal lobe, children with ASD presented significant lower FD in the structure of the left postcentral gyrus ($p = 0.01$) [24] – *see Figure 2* –, opposed to Khadem-Reza & Zare, 2022 that highlighted decreased FD in the right postcentral gyrus ($p = 0.001$) and besides that in the right superior parietal lobule ($p = 0.004$), right inferior parietal lobule ($p = 0.003$), right precuneus ($p = 0.004$), and right supramarginal gyrus ($p = 0.004$). Dona et al. 2017, in youth with autism, the functional connectivity revealed decreased H values in the left and right BA 23 ($p = 0.01$, both) – while Lai et al. 2010, in adults with ASD, had significant differences in the precuneus (BA 7 and 31), inferior parietal lobule (BA 39 and 40), and temporoparietal junction.

Related to the occipital lobe in children with ASD, Khadem-Reza & Zare, 2022 showed decreased FD in the structure of the right cuneus ($p = 0.011$), right lateral occipital sulcus ($p = 0.003$), right pericalcarine cortex ($p = 0.001$), and right lingual gyrus ($p = 0.006$). In adults with autism, only the lingual gyrus (BA 18) appeared significant lower H values on its functional connectivity [30].

Regarding temporal lobe, children with ASD showed structural significant higher FD related to the left superior temporal sulcus ($p = 0.04$) [24], opposed to Khadem-Reza & Zare, 2022 where lower FD was related to the right superior temporal sulcus ($p = 0.009$), right entorhinal cortex ($p = 0.003$), right fusiform gyrus ($p = 0.003$), right superior temporal gyrus ($p = 0.002$), right middle temporal gyrus ($p = 0.003$), right inferior temporal gyrus ($p = 0.006$), right temporal pole ($p = 0.003$), and right transverse temporal gyrus ($p = 0.002$). In Dona et al. 2017, the functional connectivity in youth with autism had decreased H values in the left hippocampus ($p = 0.02$), while in Lai et al. 2010, adults with ASD demonstrated significant differences in the temporal pole (BA 38), superior temporal gyrus (BA 22), hippocampus and parahippocampal and fusiform gyri (BA 19, 20, 35, 36, and 37), and posterior superior temporal sulcus. In addition, both children and adults with autism and comorbid ADHD manifested increased FD in the right fusiform gyrus compared to people with ASD [31].

In the limbic lobe, on Khadem-Reza & Zare, 2022, children with ASD showed structural significant decreased FD associated to the right rostral ($p = 0.006$) and right caudate ($p = 0.001$) anterior cingulate cortex, right posterior cingulate cortex ($p = 0.003$), left ($p = 0.044$) and right ($p = 0.002$) isthmus of the cingulate cortex, right insular cortex ($p = 0.012$), and left ($p = 0.047$) and right ($p = 0.014$) parahippocampal gyrus. In Lai et al. 2010, exposed that the adults with autism had lower H values in the functional connectivity of the anterior cingulate cortex (BA 32 and 33), dorsal cingulate cortex (BA 24), posterior cingulate cortex (BA 23, 29, 30, and 31), and insular cortex.

About the subcortical structures, Dona et al. 2017 presented lower H values in youth with ASD related to the functional connectivity in the left and right amygdala ($p = 0.00$ and $p = 0.01$, respectively), left and right nucleus accumbens ($p = 0.00$ and $p = 0.02$, respectively), and right caudate head ($p = 0.01$); while Lai et al. 2010 H values in adults with ASD were related to the amygdala ($p = 0.001$), basal ganglia, and thalamus. Moreover, H values in the right anterior insula were negatively correlated with ADI-R social scores and H values in the left and right retrosplenial cortex were negatively correlated with ADOS social communication scores [30].

Regarding global cerebellar structures, in children with ASD, there was a significant reduction of the FD in the right cerebellar cortex ($p = 0.006$) and, in turn, associated with cognitive abilities and with higher ADOS social affect scores ($p = 0.07$) – considering no significant group differences in cerebellar volume and normal IQ [28]. Besides, children with ASD presented flatter and less regular surface protrusions compared to neurotypical development [28]. In terms of functional connectivity, in youth with ASD, H values were significantly decreased in the left and right tuber ($p = 0.03$ and $p = 0.02$, respectively), left and right culmen ($p = 0.01$ and $p = 0.02$, respectively), right uvula ($p = 0.01$), and vermis ($p = 0.02$) – as shown in Dona et al. 2017.

Finally, in network connections, Rakshe et al. 2023 studied the FD in children with autism, showing that the connections within dorsal attention network, visual network, and cingulo-opercular network (task control) were the top dominant intra-network connections. Moreover, inter-network connections from dorsal attention to cingulo-opercular (task control) network, ventral attention to dorsal attention network, dorsal attention to somatosensory network, and auditory to visual network were dominant.

Figure S3. Laatsch et al. 2024 showing positive association between cortical irregularities and symptoms of inattention. Clusters of significant positive correlation at $p < 0.05$ after TFCE, FWE corrected combined peak-cluster level.

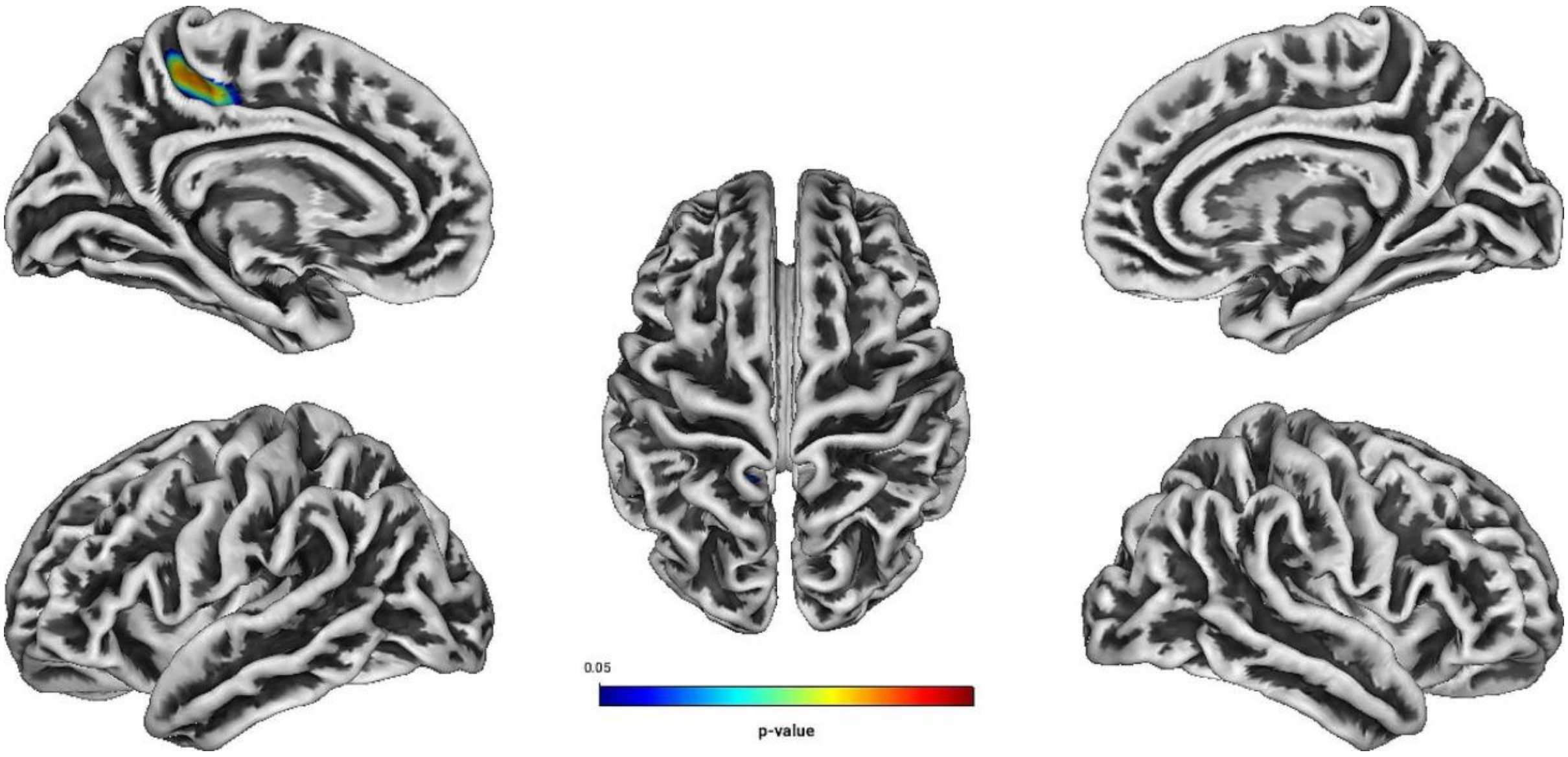

S4. Schizophrenia

To our knowledge, the fractal appearance of schizophrenia only was studied in adults and using structural MRI, although it is a condition that might begin in adolescence – *see Table 2*. Ha et al. 2005 mentioned that FD asymmetry index and logistic regression models distinguished up to 95.6% of schizophrenia from controls. Furthermore, Zhao et al. 2016 found a medium to large Cohen effect size, meaning that structural differences are visible to the naked eye of a careful observer.

Narr et al. 2004, in the first episode of schizophrenia, significant diagnosis-hemisphere interactions ($p < 0.01$) were unveiled. Notable hemispheric asymmetry was found with greater FD in the right superior frontal ($p < 0.002$), right occipital ($p < 0.01$), and right parietal ($p < 0.001$) lobes. Only in males with schizophrenia, a significant FD increase was observed in the right hemisphere compared to male controls, particularly in the superior frontal region ($p < 0.01$). Additionally, the left hemisphere superior frontal region displayed a medium effect size in FD when contrasting female groups. In contrast, Fan et al. 2025, highlighted patterns of decreased ($p < 0.001$) and increased ($p = 0.014$) morphological similarities in FD-based networks, with connections involving the default mode, frontoparietal, somatomotor, and cingulo-opercular regions – these patterns were positively correlated with the PANSS score ($p < 0.001$) and the MCCB score ($p = 0.017$).

Specifically, Nenadic et al. 2014 studied the schizophrenia profiles – i.e., predominantly negative, disorganized, and paranoid – *see Figure 3*. The predominantly negative profile showed a decreased FD associated to the frontopolar, right superior frontal, superior parietal, and left anterior cingulate cortices. The disorganized profile demonstrated FD reductions in the right and left ventrolateral prefrontal and orbitofrontal cortices, and increases in the left hemisphere – involving the inferior parietal, middle temporal, and mid-cingulate cortices. Finally, the paranoid profile presented lower FD values in the right superior parietal and left fusiform cortices, and higher values in the left posterior cingulate cortex.

About the global measures, in Sandu et al. 2008, schizophrenia had larger FD values in the gray-white matter boundary for the whole brain volume (box-count $p = 0.008$ and Minkowski $p = 0.004$) and gray-white matter boundary in the right hemisphere (box-count and Minkowski $p = 0.001$). On the other

hand, Ha et al. 2005 showed smaller FD values associated with whole brain, left and right hemispheres, and gray matter. Schizophrenia had significantly lower global FD in the right hemisphere [39, 40], while Nenadic et al. 2014 found that related to the negative and paranoid profiles, but not with the disorganized profile. Moreover, schizophrenia presented less FD values compared to controls in whole brain measures ($p < 0.01$) [44], suggesting global reductions in structural irregularity of brain. In general, anterior cerebral volume was reduced in schizophrenia and when controlling for diagnosis and sex, it was correlated with global mean FD ($p = 0.002$) [44].

Squarcina et al. 2015 demonstrated lower FD of gray matter volume, especially in frontal and temporal lobes ($p = 0.05$). Besides, lower FD irregularity was found mainly in the prefrontal cortex [40] – specifically in the left prefrontal region – and in the right frontal, left and right temporal, and right cingulate cortices [39].

Regarding the subcortical structures, Zhao et al. 2016 showed that the gray matter surface had a significant reduction of FD in the left ($p < 0.001$) and right ($p = 0.004$) hippocampus, as well as left thalamus ($p = 0.005$) in schizophrenia compared to control.

S5. Overview of fractal neuroimaging findings across diverse psychiatric diagnoses, including region-specific alterations and clinical correlations

Several studies show imbalances in their samples, such as low female representation in autism, reliance on healthy volunteers in anxiety, or absence of males in eating disorders, among others. These characteristics reflect what is often seen in clinical practice and society, but they also underline the need for more diverse and representative samples in future research. Below is a brief summary of the findings in each respective sample. For specific details, please refer to each study in its respective table.

Neurodevelopmental Disorders

Fractal studies on ASD, primarily involving male youths, reveal distinct structural and functional anomalies in the frontal lobe with consistently reduced FD [24, 26, 27, 30]. Lower FD values, specifically in the frontal and parietal areas, may correlate with cognitive and social impairments since functional connectivity analyses link these findings to clinical scores [24, 29-31]. Fractal analyses also revealed altered values in regions such as the amygdala, retrosplenial cortex, and temporal lobes, relating these changes to symptomatology, including language deficits and difficulties in social interaction [24].

ADHD studies, though limited, suggest reduced FD in the prefrontal cortex along with asymmetry patterns [32]. These findings align with known deficits in executive function in ADHD populations. However, FD differences appear less pronounced in adults [33, 34], potentially reflecting the development of compensatory mechanisms over time.

Psychotic Disorders

FD studies in schizophrenia, predominantly in adults, exposed reduced fractality in frontal, temporal, and subcortical regions [36-38, 40, 43]. Structural abnormalities informed robust evidence, even on the first episode [43]. Subtypes of schizophrenia displayed distinct fractal profiles [38] and asymmetric patterns aligned with clinical presentations, particularly negative symptoms.

Bipolar disorder highlighted increased global FD values on structure [45]. Regionally, this condition was marked by both increased and decreased FD in specific brain regions [37, 45], potentially reflecting neuropathological mechanisms such as hyperconnectivity or compensatory processes.

Mood and Anxiety Disorders

Although mood disorders are the most prevalent in society, they have been rarely focused on fractal analysis studies. Anxiety studies focused on functional connectivity, highlighting positive correlations between fractal values and emotional regulation scores [48-50] – including prefrontal-limbic regions [49, 50]. Depression, despite its prevalence, lacks substantial fractal analyses, presenting an avenue for future exploration.

Trauma- and Stressor-Related Disorders

PTSD studies revealed bilateral reductions in cortex FD across frontal, parietal, and temporal regions [51]. These alterations correlated with symptom severity, aligning with the dysregulation of stress-related neural networks. Sex-specific findings, particularly in the hippocampus and anterior cingulate cortex, underscore the influence of sex on structural brain changes.

Other Disorders

Anorexia nervosa studies demonstrated reduced FD in global and regional measures, varying between acute and recovered states [53, 54]. These results may reflect acute effects of malnutrition and recovery-related neuroplasticity.

Drug users' studies showed FD reductions in cortical regions, such as the left insula and orbitofrontal cortex [58, 61]. These alterations align with addiction profiles and disruptions in reward-processing networks.

Table S6. Characterization of psychiatric conditions according to the fractal pattern of the brain regions.

BRAIN REGION	REFERENCES	PSYCHIATRIC CONDITION	SPECIFIC ROIs	FRACTAL PATTERN
Frontal lobe	Arutiunian et al., 2023<br>Khadem-Reza & Zare, 2022<br>Yamagata et al., 2019<br>Lai et al., 2010	ASD	Orbitofrontal cortex	· Lower structural FD<br>· Negatively correlated with AQ attention to detail score
			Medial prefrontal cortex	Lower functional H values
			Superior frontal gyrus	Lower structural FD
			Middle frontal gyrus	· Lower structural FD<br>· Negatively correlated with AQ attention to detail score
			Inferior frontal gyrus	Lower functional H values
	Li et al., 2007	ADHD	Left prefrontal cortex	Lower structural FD
	Squarcina et al., 2015<br>Nenadic et al., 2014<br>Yotter et al., 2011<br>Yotter et al., 2010<br>Narr et al., 2004	Schizophrenia	Orbitofrontal cortex	Lower structural FD in disorganized profile
			Right frontal cortex	Lower structural FD
			Prefrontal cortex	Lower structural FD:<br>· left region in general schizophrenia<br>· ventrolateral region in disorganized profile
			Right superior frontal cortex	· Higher FD in first episode<br>· Lower FD in predominantly negative profile
	Guo et al,. 2024<br>Nenadic et al., 2017<br>Squarcina et al., 2015	Bipolar disorder	Left lateral and right orbitofrontal cortex	· Higher structural FD<br>· Higher functional H values
			Left superior and inferior frontal gyrus	Higher functional H values
			Right caudate middle frontal gyrus	Lower structural FD
	Mujica-Parodi et al., 2014<br>Olejarczyk, 2007	Anxiety state	Dorsolateral prefrontal cortex	Significant differences
			Left inferior frontal gyrus	· Lower functional FD<br>· Positively correlated with regulatory control
	Kritikos et al., 2021	PTSD	Frontal cortex	Lower structural FD
	Nickel et al., 2019	Anorexia nervosa (acute)	Left precentral gyrus	Higher structural FD
	Ghosh et al., 2024	Heroin addiction	Left transverse frontopolar gyrus and sulcus	Higher structural FD
			Right middle frontal gyrus	Higher structural FD
			Left lateral orbital gyrus	Higher structural FD
	Trevisan et al., 2023	Cocaine addiction	Left and medial orbitofrontal cortex	· Lower fractality<br>· Medial region positively correlated with the age of onset and negatively correlated with the BIS score
Temporal lobe	Chen et al., 2023<br>Khadem-Reza & Zare, 2022<br>Lai et al., 2010	ASD	Superior temporal gyrus	Lower structural FD
			Fusiform gyrus	Lower structural FD<br>· Significant differences in functional<br>· Higher functional FD in ASD with comorbid ADHD
			Right enthorhinal cortex	Lower structural FD
	Squarcina et al., 2015<br>Nenadic et al., 2014<br>Yotter et al., 2011	Schizophrenia	Temporal cortex	Lower structural FD
			Left middle temporal cortex	Higher structural FD in disorganized profile
			Left fusiform cortex	Lower structural FD in paranoid profile
	Guo et al., 2024<br>Nenadic et al., 2017	Bipolar disorder	Left fusiform cortex	Lower structural FD
			Entorhinal cortex	Lower structural FD
			Left inferior temporal gyrus	Higher functional H values
	Kritikos et al., 2021	PTSD	Temporal cortex	Lower structural FD
	Weber et al., 2014	Ethanol intoxication	Right superior temporal gyrus	Higher functional H values
Limbic lobe	Khadem-Reza & Zare, 2022<br>Lai et al., 2010	ASD	Anterior cingulate cortex	· Lower structural FD<br>· Lower functional H values
			Dorsal cingulate cortex	Lower functional H values
			Posterior cingulate cortex	· Lower structural FD<br>· Lower functional H values
			Insular cortex	· Lower functional H values<br>· Right anterior region negatively correlated with ADI-R social score

	Nenadic et al., 2014<br>Yotter et al., 2011	Schizophrenia	Cingulate cortex	Lower FD:<br>· right region in general schizophrenia<br>· left anterior region in predominantly negative profile<br>· middle region in disorganized profile<br>· left posterior region in paranoid profile
	Guo et al., 2024<br>Nenadic et al., 2017	Bipolar disorder	Posterior cingulate cortex	Lower structural FD
			Right insular cortex	Higher functional H values
	Gentili et al., 2015	Anxiety state	Posterior cingulate cortex	· Higher fractality<br>· Positively correlated with fear of negative evaluation
	Vatheuer et al., 2021	PTSD	Anterior cingulate cortex	Significant differences
	Trevisan et al., 2023	Cocaine addiction	Left insular cortex	Lower fractality
	Weber et al., 2014	Ethanol intoxication	Left posterior cingulate cortex	Higher functional H values
			Right insular cortex	Higher functional H values
Parietal lobe	Khadem-Reza & Zare, 2022	ASD	Precuneus	· Lower structural FD<br>· Lower functional H values
			Right superior parietal lobule	Lower structural FD
			Inferior parietal lobule	· Lower structural FD<br>· Lower functional H values
			Right supramarginal gyrus	Lower structural FD
	Laatsch et al. 2024	ADHD	Precuneus (left hemisphere)	Positive correlation with symptoms of inattention
	Nenadic et al., 2014<br>Narr et al., 2004	Schizophrenia	Right parietal cortex	Higher structural FD in first episode
			Superior parietal cortex	Lower structural FD in predominantly negative and paranoid profiles
			Inferior parietal cortex	Lower structural FD in disorganized profile
	Guo et al,. 2024<br>Nenadic et al., 2017	Bipolar disorder	Right precuneus	Higher structural FD
			Right inferior parietal lobule	Higher functional H values
	Gentili et al., 2015	Anxiety state	Precuneus	Functional H values positively correlated with BFNE and LSAS scores
	Kritikos et al., 2021	PTSD	Parietal cortex	Lower structural FD
	Collantoni et al., 2020	Anorexia nervosa (acute and recovered)	Parietal cortex	Lower structural FD
	Trevisan et al., 2023	Cocaine addiction	Left supramarginal gyrus	Lower fractality
	Vatheuer et al., 2021	BPD	Right superior parietal cortex	Higher structural FD
Subcortical structures	Dona et al., 2017<br>Lai et al., 2010	ASD	Hippocampus	Lower functional H values
			Parahippocampus	Significant differences
			Amygdala	Lower functional H values
			Thalamus	Significant differences
	Zhao et al., 2016	Schizophrenia	Hippocampus	Lower functional FD
			Left thalamus	Lower functional FD
	Gentili et al., 2015<br>Mujica-Parodi et al., 2014<br>Olejarczyk, 2007	Anxiety state	Hippocampus	Significant differences
			Right parahippocampus	Functional H values positively correlated with LSAS scores
			Left amygdala	Functional H values positively correlated with regulatory control
	Vatheuer et al., 2021	PTSD	Hippocampus	Significant differences
			Amygdala	Significant differences
	Weber et al., 2014	Ethanol intoxication	Thalamus	Higher functional H values

ADHD Attention-Deficit/Hyperactivity Disorder; AQ Autism Quotient; ASD Autism Spectrum Disorder; BFNE Brief Fear of Negative Evaluation Scale; BPD Borderline Personality Disorder; FD Fractal Dimension; LSAS Liebowitz Social Anxiety Scale; PTSD Post-Traumatic Stress Disorder.
